# Iron status and mental disorders: A Mendelian randomization study

**DOI:** 10.3389/fnut.2022.1084860

**Published:** 2022-12-15

**Authors:** Jiaqi Qiu, Fuzhi Lian, Xuexian Fang

**Affiliations:** ^1^Department of Nutrition and Toxicology, School of Public Health, Hangzhou Normal University, Hangzhou, China; ^2^Key Laboratory of Elemene Class Anti-Cancer Chinese Medicines of Zhejiang Province, Hangzhou Normal University, Hangzhou, China

**Keywords:** iron, ferritin, transferrin, mental disorders, UK Biobank, Mendelian randomization

## Abstract

**Background:**

Mental disorders account for an enormous global burden of disease, and has been associated with disturbed iron metabolism in observational studies. However, such associations are inconsistent and may be attributable to confounding from environmental factors. This study uses a two-sample Mendelian randomization (MR) analysis to investigate whether there is any causal effect of systemic iron status on risk of 24 specific mental disorders.

**Methods:**

Genetic variants with concordant relations to 4 biomarkers of iron status (serum iron, ferritin, transferrin saturation, and transferrin) were obtained from a genome-wide association study performed by the Genetics of Iron Status (GIS) consortium. Summary-level data for mental disorders were obtained from the UK Biobank. An inverse-variance weighted (IVW) approach was used for the main analysis, and the simple median, weighted median and MR-Egger methods were used in sensitivity analyses.

**Results:**

Genetically predicted serum iron, ferritin, and transferrin saturation were positively associated with depression and psychogenic disorder, and inversely associated with gender identity disorders. A higher transferrin, indicative of lower iron status, was also associated with increased risk of gender identity disorders and decreased risk of psychogenic disorder. Results were broadly consistent when using multiple sensitivity analyses to account for potential genetic pleiotropy.

**Conclusion:**

Our findings offer a novel insight into mental health, highlighting a detrimental effect of higher iron status on depression and psychogenic disorder as well as a potential protective role on risk of gender identity disorders. Further studies regarding the underlying mechanisms are warranted for updating preventative strategies.

## Introduction

The essential element iron is closely involved in diverse fundamental biological processes, such as energy metabolism, oxygen transport, redox balance, and immunological modulation (1). Iron deficiency causes a number of pathological consequences, the most prominent being anemia. More than 2 billion individuals suffer from iron deficiency anemia worldwide, which may contribute to cognitive developmental defects in children (2). However, iron overload is also common and equally detrimental, as excess labile iron catalyzes the generation of reactive oxygen species (ROS) *via* the Fenton reaction and can trigger non-apoptotic cell death (3).

Disturbed iron homeostasis is related to a wide variety of chronic diseases, including mental disorders. In specific brain regions of patients with Alzheimer’s disease (AD), significant iron accumulation has been clearly observed and correlated well with a decline in cognitive function (4, 5). Increased iron concentrations were also reported as characteristic of the degenerating substantia nigra of Parkinson’s disease (PD), the second most prevalent neurodegenerative disease after AD (6). However, limited epidemiological evidence on the relationship between iron status and risk of other mental disorders is available.

Here, we conducted a Mendelian randomization (MR) study to robustly evaluate whether systemic iron status affects risk of multiple mental disorders. By exploiting genetic variants as instrumental variables of systemic iron status, the approach greatly avoided the risk of residual confounding as genetic variants are randomly allocated at conception and therefore irrelevant to environmental factors (7). Since disease development typically cannot alter the germline nuclear sequences, it may also overcome the problem of reverse causation (8). In this way, we employ MR to investigate the causal role for iron status in mental disorders, which would have important public health implications.

## Materials and methods

In this present two-sample MR study, we used data from two different genome-wide association studies (GWASs)—one for the exposures and one for the outcome—to estimate the effects of exposure on outcome. Only summary-level data from these published studies were used. Appropriate patient consent and ethical approval were obtained in the original studies.

### Genetic instrument selection

For unbiased detection of causal effects, the genetic variants used as genetic variants in this MR analysis must satisfy three key assumptions: (1) the used genetic variants should be robustly associated with iron status, (2) the used should not be associated with any confounders and (3) the genetic variants should influence the outcome (risk of specific mental disorder) only through iron status, rather than alternative pathways. The second and third assumptions are collectively known as independence from pleiotropy.

A large-scale meta-analysis of GWASs by the Genetics of Iron Status (GIS) Consortium was used to obtain single nucleotide polymorphisms (SNPs) proposed to be associated with systemic iron status. Data from 11 discovery cohorts and 8 replication cohorts were used in the meta-analysis, consisting of 48,972 individuals of European ancestry. It identified that 11 genome-wide-significant SNPs related to biomarkers of iron status, including increased serum iron, ferritin, transferrin saturation and decreased transferrin. Of these, three SNPs [rs1800562 and rs1799945 in the hemochromatosis (HFE) gene and rs855791 in the transmembrane serine protease 6 (TMPRSS6) gene] were robustly associated with concordant variance for each biomarker (Supplementary Table 1) (9). In addition, these SNPs are not in linkage disequilibrium and have been used as instrumental variables for systemic iron status in previous MR studies (10–12). Therefore, we included them in our MR analysis to predict systemic iron status.

*F* statistic of first-stage regression was employed to evaluate the strength of the instruments and was calculated using the following equation: *F* = (*R*^2^/*k*)/([1-*R*^2^]/[*n-k*-1]), where *R*^2^ is the proportion of the iron status variability accounted for by the SNP, *k* is the number of instruments used in the model and *n* is the sample size (13). To minimize weak instrument bias, only SNPs with an *F* statistic > 10 were included in subsequent analyses.

### Outcome data sources

Summary-level GWAS statistics for 24 specific mental disorders were extracted from the Pan-ancestry genetic analysis of the UK Biobank (14). This UK-based prospective cohort study was approved by the North West Multicentre Research Ethics Committee, and recruited around 500,000 genotyped adults aged 40–69 years. Detailed characteristics of cases and controls can be found in Supplementary Table 2. The summary-level data are publicly available at https://pan.ukbb.broadinstitute.org.

### Statistical analysis

An inverse-variance weighted (IVW) method with random-effects was used to generate the main MR estimates for the causal associations between each measure of iron status and risk of mental disorders. A threshold of *P* < 0.05 was used to determine statistical significance. Odds ratios (ORs) with 95% confidence intervals (CIs) of mental disorders are per one standard deviation (SD) increase in genetically predicted serum iron, log10 ferritin, transferrin saturation and transferrin levels. Cochran’s *Q* test was applied to evaluate heterogeneity in IVW estimates across different instruments (interpreting *P* < 0.05 as evidence of heterogeneity) (15).

Pleiotropy refers to a genetic variant influencing the outcome of interest through pathways independent of the risk factor. We then conducted a range of sensitivity analyses, including the simple median, weighted median and MR-Egger methods, to address potential pleiotropy. The simple and weighted median methods provide consistent MR estimates even when up to 50% of the information comes from invalid instrumental variables (16). The MR-Egger method is able to assess whether genetic variants have pleiotropic effects on the outcome and provides a consistent estimate of the causal effect even if all genetic variants are invalid (17). Since the relatively low statistical power of these methods compared with the main IVW analysis, they were used solely to confirm a consistent effect estimate, rather than to ascertain statistical significance itself *via* a *p*-value threshold. We also searched the PhenoScanner database^1^ to detect secondary phenotypes for the SNPs associated with the iron status biomarkers (18).

All MR analyses were performed using the TwoSampleMR package by R 4.0.2 software.^2^

## Results

Three SNPs including rs1800562 and rs1799945 in the *HFE* gene and rs855791 in the *TMPRSS6* gene were employed for our main analysis. The *F* statistics for the three SNPs ranged from 47 for 2,127 across all four biomarkers of systematic iron status, as described previously (10, 12), making significant bias from use of weak instruments unlikely (13).

**FIGURE 1:**
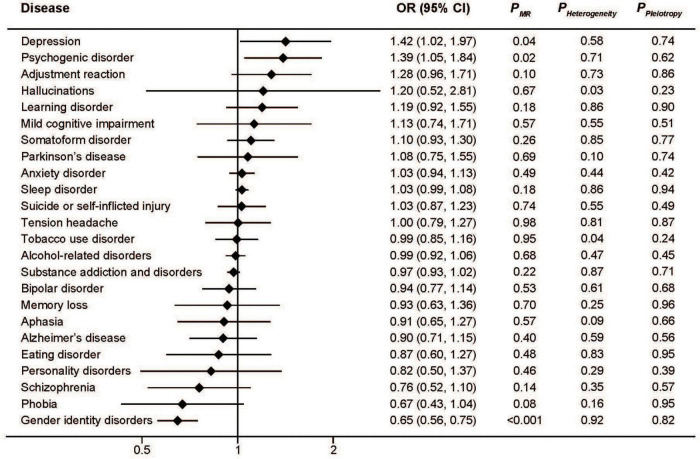
Associations between genetically predicted serum iron and risk of 24 specific mental disorders.

Genetically predicted high serum iron levels were positively associated with depression (OR: 1.41; 95%CI: 1.02, 1.97; *P* = 0.04) and psychogenic disorder (OR: 1.39; 95%CI: 1.05, 1.84; *P* = 0.02), but inversely associated with gender identity disorders (OR: 0.65; 95%CI: 0.56, 0.75; *P* < 0.001). No association was found in the other 21 mental disorders. We detected significant heterogeneity only in the analyses of hallucinations (*P* = 0.03) and tobacco use disorder (*P* = 0.04). Pooled MR estimates for the effect of increased serum iron on risk of different mental disorders are shown graphically in Figure 1.

**FIGURE 2:**
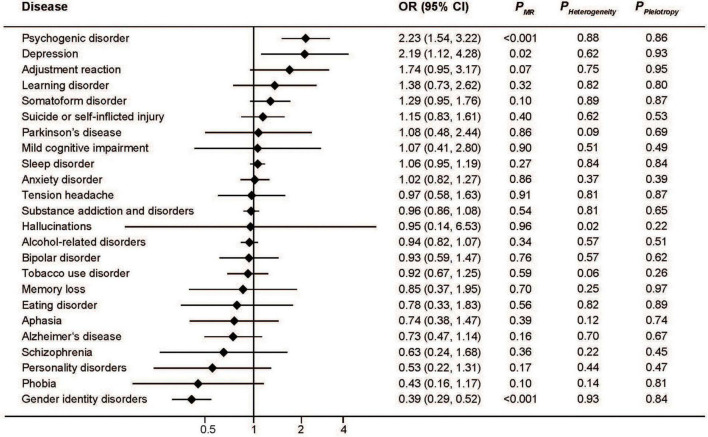
Associations between genetically predicted serum ferritin and risk of 24 specific mental disorders.

**FIGURE 3:**
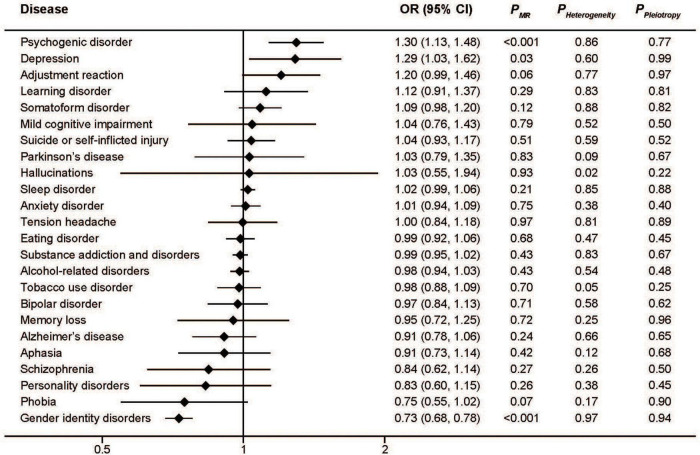
Associations between genetically predicted transferrin saturation and risk of 24 specific mental disorders.

Similar to serum iron, the MR analysis showed a protective effect of ferritin on gender identity disorders as well as a detrimental effect on depression and psychogenic disorder (Figure 2). The pooled ORs for per SD unit increase of log_10_ (ferritin) on psychogenic disorder, depression, and gender identity disorders were 2.23 (95%CI: 1.54, 3.22; *P* < 0.001), 2.19 (95%CI: 1.12, 4.28; *P* = 0.02), and 0.39 (95%CI: 0.29, 0.52; *P* < 0.001), respectively. Consistent associations were also found with transferrin saturation (Figure 3). The pooled ORs for per SD unit increase of transferrin on psychogenic disorder, depression, and gender identity disorders were 1.30 (95%CI: 1.13, 1.48); *P* < 0.001, 1.29 (95%CI: 1.03, 1.62; *P* = 0.03), and 0.73 (95%CI: 0.68, 0.78; *P* < 0.001), respectively.

**FIGURE 4:**
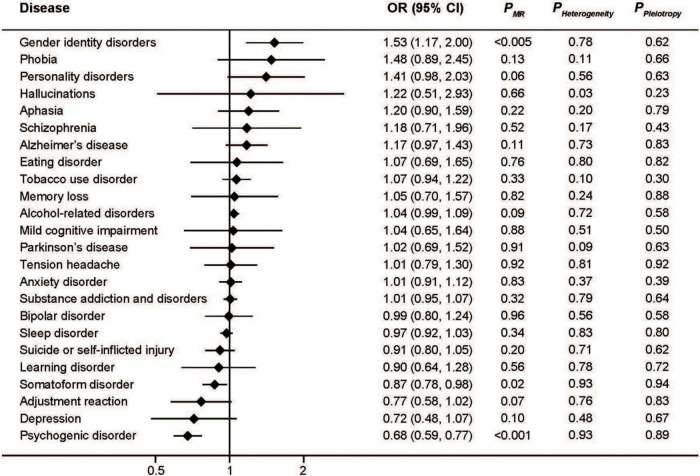
Associations between genetically predicted serum transferrin and risk of 24 specific mental disorders.

In addition, higher transferrin levels, which are indicative of lower iron status, were associated with increased risk of gender identity disorders (OR: 1.53; 95%CI: 1.17, 2.00; *P* < 0.005) as well as decreased risk of somatoform disorder (OR: 0.87; 95%CI: 0.78, 0.98; *P* = 0.02) and psychogenic disorder (OR: 0.68; 95%CI: 0.59, 0.77; *P* < 0.001) (Figure 4). The effect of transferrin on depression seemed protective, but did not reach statistical significance (OR: 0.72; 95%CI: 0.48, 1.07; *P* = 0.10).

The MR-Egger approach did not produce evidence of pleiotropy in any of our analyses (Figures 1–4). We further examined potential pleiotropy using the PhenoScanner database. A potentially pleiotropic effect on risk of these mental disorders may be contributed by rs1800562 because of its association with reduced total and low-density lipoprotein cholesterol levels (19). Moreover, sensitivity analyses using simple median, weighted median, and MR Egger methods produced directionally consistent effects as the IVW estimates, supporting the consistency and robustness of our findings (Table 1).

**TABLE 1:** Sensitivity analyses of the associations between genetically predicted iron status and mental disorders.

Disease/methods	Serum iron			Ferritin			Transferrin saturation			Transferrin		
	β	*SE*	*P*	β	*SE*	*P*	β	*SE*	*P*	β	*SE*	*P*
**Alzheimer’s disease**												
Weighted median	-0.12	0.20	0.54	-0.40	0.40	0.34	-0.12	0.13	0.36	0.17	0.18	0.35
Simple median	-0.08	0.20	0.70	-0.26	0.52	0.64	-0.08	0.17	0.66	0.20	0.39	0.60
MR-Egger	-0.65	0.69	0.52	-0.64	0.68	0.52	-0.24	0.26	0.53	0.20	0.24	0.55
**Aphasia**												
Weighted median	-0.14	0.14	0.31	-0.32	0.27	0.24	-0.12	0.090	0.20	0.19	0.12	0.10
Simple median	-0.24	0.15	0.12	-0.38	0.30	0.17	-0.14	0.10	0.17	0.16	0.15	0.28
MR-Egger	-0.59	0.84	0.61	-0.59	0.82	0.60	-0.25	0.31	0.57	0.24	0.26	0.53
**Mild cognitive impairment**												
Weighted median	0.13	0.34	0.71	0.0021	0.64	1.00	0.031	0.21	0.88	0.034	0.28	0.90
Simple median	0.22	0.34	0.51	0.73	0.99	0.43	0.21	0.29	0.47	-0.89	0.75	0.23
MR-Egger	-0.90	1.10	0.56	-0.89	1.09	0.56	-0.33	0.42	0.57	0.28	0.38	0.59
**Memory loss**												
Weighted median	-0.16	0.20	0.42	-0.24	0.41	0.55	-0.08	0.14	0.54	0.03	0.18	0.85
Simple median	-0.12	0.21	0.55	-0.20	0.44	0.65	-0.07	0.14	0.63	0.08	0.27	0.75
MR-Egger	-0.14	1.10	0.92	-0.13	1.09	0.93	-0.03	0.42	0.96	-0.0010	0.38	1.00
**Hallucinations**												
Weighted median	0.53	0.33	0.11	-0.08	0.58	0.88	0.05	0.19	0.79	0.25	0.24	0.29
Simple median	0.52	0.37	0.16	1.52	1.12	0.17	0.43	0.32	0.18	-0.87	0.78	0.26
MR-Egger	-2.18	0.93	0.26	-2.16	0.92	0.26	-0.84	0.36	0.26	0.75	0.32	0.26
**Schizophrenia**												
Weighted median	-0.19	0.22	0.37	-0.30	0.44	0.50	-0.12	0.15	0.42	0.14	0.20	0.47
Simple median	-0.23	0.23	0.32	-0.77	0.71	0.28	-0.22	0.21	0.28	0.96	0.61	0.11
MR-Egger	0.37	0.84	0.74	0.35	0.84	0.75	0.12	0.33	0.78	-0.09	0.31	0.82
**Bipolar disorder**												
Weighted median	-0.03	0.16	0.86	0.003	0.34	0.99	0.001	0.11	0.99	-0.03	0.15	0.85
Simple median	0.03	0.18	0.87	0.045	0.44	0.91	0.016	0.13	0.90	-0.02	0.26	0.94
MR-Egger	0.25	0.57	0.74	0.25	0.56	0.73	0.11	0.22	0.71	-0.11	0.20	0.69
**Depression**												
Weighted median	0.47	0.25	0.06	0.83	0.56	0.11	0.29	0.18	0.10	-0.31	0.23	0.18
Simple median	0.46	0.28	0.10	0.79	0.64	0.22	0.28	0.20	0.15	-0.34	0.35	0.33
MR-Egger	0.73	0.91	0.57	0.71	0.90	0.58	0.26	0.35	0.60	-0.21	0.33	0.64
**Suicide or self-inflicted injury**												
Weighted median	0.009	0.13	0.95	0.15	0.26	0.55	0.04	0.09	0.66	-0.09	0.12	0.44
Simple median	-0.04	0.14	0.79	-0.13	0.39	0.74	-0.04	0.12	0.75	0.16	0.28	0.56
MR-Egger	0.49	0.45	0.48	0.48	0.45	0.48	0.18	0.17	0.48	-0.16	0.16	0.49
**Phobia**												
Weighted median	-0.33	0.21	0.11	-0.62	0.41	0.10	-0.22	0.14	0.11	0.29	0.18	0.12
Simple median	-0.40	0.22	0.07	-0.65	0.50	0.19	-0.23	0.15	0.13	0.38	0.50	0.46
MR-Egger	-0.50	1.28	0.76	-0.52	1.26	0.75	-0.22	0.48	0.72	0.23	0.41	0.68
**Personality disorders**												
Weighted median	-0.16	0.29	0.58	-0.71	0.56	0.21	-0.21	0.17	0.24	0.40	0.25	0.11
Simple median	-0.04	0.31	0.90	-0.13	0.86	0.88	-0.04	0.25	0.88	0.16	0.55	0.77
MR-Egger	-1.50	0.94	0.36	-1.47	0.93	0.36	-0.56	0.36	0.36	0.48	0.32	0.38
**Gender identity disorders**												
Weighted median	-0.51	0.29	0.08	-0.89	0.61	0.14	-0.30	0.20	0.14	0.41	0.28	0.13
Simple median	-0.51	0.32	0.11	-1.00	0.79	0.20	-0.30	0.23	0.20	0.85	0.59	0.15
MR-Egger	-0.72	1.05	0.62	-0.72	1.04	0.61	-0.28	0.40	0.61	0.26	0.36	0.60
**Psychogenic disorder**												
Weighted median	0.41	0.28	0.14	0.82	0.57	0.15	0.29	0.18	0.12	-0.38	0.25	0.40
Simple median	0.42	0.30	0.16	0.85	0.70	0.22	0.30	0.21	0.16	-0.36	0.51	0.47
MR-Egger	0.98	0.97	0.50	0.97	0.96	0.49	0.39	0.37	0.49	-0.36	0.33	0.48
**Somatoform disorder**												
Weighted median	0.14	0.25	0.58	0.28	0.49	0.57	0.10	0.16	0.53	-0.14	0.23	0.53
Simple median	0.19	0.26	0.47	0.30	0.63	0.63	0.11	0.18	0.56	-0.13	0.43	0.77
MR-Egger	0.41	0.85	0.72	0.41	0.85	0.72	0.16	0.33	0.70	-0.16	0.29	0.69
**Adjustment reaction**												
Weighted median	0.24	0.31	0.44	0.46	0.62	0.46	0.16	0.20	0.43	-0.25	0.28	0.37
Simple median	0.30	0.33	0.36	0.48	0.76	0.53	0.17	0.24	0.48	-0.21	0.60	0.73
MR-Egger	0.48	1.07	0.73	0.48	1.05	0.73	0.20	0.41	0.71	-0.20	0.37	0.68
**Eating disorder**												
Weighted median	-0.18	0.49	0.71	-0.25	1.08	0.82	-0.086	0.34	0.80	0.04	0.47	0.93
Simple median	-0.12	0.55	0.83	-0.19	1.32	0.89	-0.07	0.38	0.86	0.08	0.93	0.93
MR-Egger	0.0013	1.83	1.00	0.018	1.81	0.99	0.03	0.70	0.98	-0.05	0.63	0.95
**Tension headache**												
Weighted median	-0.09	0.30	0.77	-0.14	0.59	0.81	-0.05	0.21	0.81	0.02	0.27	0.95
Simple median	-0.08	0.31	0.79	-0.13	0.78	0.86	-0.05	0.23	0.84	0.06	0.49	0.91
MR-Egger	-0.21	1.04	0.87	-0.20	1.03	0.88	-0.07	0.40	0.90	0.04	0.36	0.92
**Learning disorder**												
Weighted median	0.18	0.37	0.62	0.26	0.80	0.75	0.09	0.25	0.72	-0.07	0.35	0.84
Simple median	0.13	0.40	0.75	0.20	1.03	0.84	0.07	0.29	0.81	-0.09	0.64	0.89
MR-Egger	-0.04	1.37	0.98	-0.20	1.03	0.88	-0.03	0.53	0.96	0.05	0.47	0.94
**Substance addiction and disorders**												
Weighted median	-0.03	0.07	0.64	-0.04	0.14	0.79	-0.02	0.05	0.73	0.007	0.06	0.92
Simple median	-0.04	0.08	0.56	-0.14	0.19	0.46	-0.04	0.06	0.49	0.11	0.15	0.43
MR-Egger	0.09	0.24	0.78	0.09	0.24	0.78	0.03	0.09	0.79	-0.03	0.08	0.80
**Alcohol-related disorders**												
Weighted median	-0.01	0.05	0.82	-0.09	0.10	0.36	-0.03	0.03	0.40	0.05	0.04	0.30
Simple median	-0.01	0.06	0.83	-0.03	0.13	0.80	-0.01	0.04	0.81	0.02	0.09	0.82
MR-Egger	-0.20	0.16	0.44	-0.20	0.16	0.44	-0.08	0.06	0.43	0.07	0.06	0.43
**Tobacco use disorder**												
Weighted median	0.02	0.06	0.68	-0.08	0.11	0.46	-0.01	0.03	0.70	0.07	0.04	0.13
Simple median	0.07	0.06	0.30	0.22	0.19	0.26	0.06	0.05	0.24	-0.18	0.13	0.19
MR-Egger	-0.43	0.17	0.25	-0.42	0.17	0.25	-0.16	0.07	0.25	0.14	0.06	0.25
**Sleep disorder**												
Weighted median	0.02	0.07	0.80	0.03	0.14	0.82	0.01	0.05	0.81	-0.02	0.06	0.80
Simple median	0.02	0.07	0.78	0.03	0.18	0.86	0.01	0.05	0.83	-0.03	0.14	0.81
MR-Egger	0.01	0.25	0.98	0.01	0.25	0.98	0.01	0.10	0.96	-0.01	0.09	0.94
**Parkinson’s disease**												
Weighted median	0.05	0.15	0.73	0.00070	0.30	1.00	0.0017	0.10	0.99	0.05	0.13	0.68
Simple median	-0.04	0.17	0.83	-0.06	0.33	0.86	-0.02	0.11	0.85	0.03	0.18	0.89
MR-Egger	-0.34	0.96	0.79	-0.35	0.95	0.78	-0.15	0.36	0.75	0.16	0.31	0.70

## Discussion

Mental disorders affected more than 1 billion people globally and lead to 19% of all years lived with disability (20). Observational epidemiological studies have associated altered iron levels with risk of AD or PD; however, these studies are susceptible to confounding and reverse causation, and thus it remains unclear whether these connections are true. More importantly, the effect of iron on most other mental disorders remains unknown. Therefore, it is necessary to better understand the causal relationship between iron status and mental disorders in humans, in order to provide evidence to support further preclinical or clinical studies.

Iron is generally assumed to be a risk factor for AD, in line with the well-known phenomenon of iron accumulation in specific brain regions of AD patients (4), but a number of epidemiological studies have questioned this conclusion (21–24). In this study, our results showed no causal association between genetically determined markers of systemic iron status and AD. One explanation for the lack of clear relationship between serum iron status and cognitive decline is that circulating iron/ferritin levels may not be a reliable indicator of brain iron in AD patients (25).

One previous MR study indicated that increased serum iron levels are causally associated with a decreased risk of developing PD (26). Epidemiological studies, however, provided strong evidence about significantly higher serum iron levels in PD patients than health controls (27). Our study failed to replicate any of these findings, as no significant associations between iron biomarkers and PD risk were found. These conflicting results illustrate the complexity of the problem, and more investigation is required.

Our study increases the probability that iron overload has a clinically relevant impact on the risk of depression. This finding is important since evidence on the association between serum iron levels and depression risk collected so far has been controversial. Iron deficiency was generally thought of as a risk factor for depression. Stewart and Hirani once studied about 2,000 elderly individuals, and found that anemia and low serum ferritin levels were linked with depressive symptoms (28). A meta-analysis also indicated that dietary iron intake is inversely associated with risk of depression, but its result is difficult to interpret owing to limited number of included studies and high heterogeneity (29). However, recent studies have shown effects of iron in opposite directions (30, 31). These epidemiological studies suffer from reverse causation, and their conclusions may be confounded by disease status. For instance, inflammation influences iron metabolism resulting in an increase in serum ferritin and a decrease in serum iron at the same time. Given neuroinflammation has been considered to impact the development of depression (32), it may drive both higher iron status and greater depression risk. Based on MR approach, we have used genetic variants as instrumental variables for iron status to overcome these limitations of observational studies and strengthen the evidence for a detrimental effect of iron status on depression risk. We also found that genetically high iron status was associated with higher risk of psychogenic disorder and lower risk of gender identity disorders. To the best of our knowledge, this is the first evidence linking iron status to these mental disorders.

There are very few studies investigating the molecular mechanisms involved in psychogenic disorder as well as gender identity disorders. And there’s still no direct evidence that shows exactly how iron metabolism affects risk of these mental disorders. However, brain capture of blood iron are necessary for an appropriate synthesis of neurotransmitters, such as serotonin, dopamine, and noradrenaline. In addition, these neurotransmitters, involved in emotional behaviors, depend on neuron aromatic hydoxylases functioning with iron as essential cofactor (33). Iron deficient animals had significantly lower dopamine D2 receptor densities in the frontal cortex and caudate putamen. Meanwhile, serotonin (5-HT) transporter densities increased in the nucleus accumbens (34). Noradrenaline also has impact on neuroplasticity *via* brain-derived neurotrophic factor, which is key for prefrontal and hippocampus neurons playing a role in depression (35). Thus, abnormality of iron metabolism may cause changes in these mental disorders by affecting neurotransmitters. More population studies are needed to confirm the association; meanwhile, further mechanism studies are warranted to examine the biological connection between iron homeostasis and the diseases.

Key strengths of the study are primarily attributed to the MR design, which overcomes many unavoidable limitations introduced in observational studies. In addition, we systematically assessed the associations between four individual iron biomarkers and 24 mental disorders using summary-level data from large-scale genetic consortia and cohorts. Another strength is that the consistency between different MR approaches suggests the robustness of our findings.

There are also several limitations to this study. First, the statistical power was low in several analyses due to limited case number for some types of mental disorders. And it is also hard to perform stratified analyses by gender, age, or other categories. In addition, potential pleiotropy could not be completely ruled out. Although no evidence was found by the MR-Egger method, residual bias may still exist because the exact biological function of the SNPs associated with iron status is not entirely clear. Last, we investigated the association between iron status within the normal range and health risk. Therefore, our findings may not be used to make inferences regarding the effect of pathological iron overload caused by primary or secondary hemochromatosis.

In summary, the present two-sample MR study is the first to systematically evaluate the causal role of iron status for a wide range of mental disorders. Our findings may highlight the potential therapeutic targets and preventative strategies. Future research is required to examine the causal associations in various populations with different ethnic backgrounds based on individual-level data, as well as the possible underlying mechanisms.

## Data availability statement

The original contributions presented in this study are included in the article/Supplementary material, further inquiries can be directed to the corresponding author.

## Ethics statement

The studies involving human participants were reviewed and approved by the Ethics Committee of Hangzhou Normal University. The patients/participants provided their written informed consent to participate in this study.

## Author contributions

XF designed the research and wrote the manuscript. JQ and FL performed the statistical analyses. All authors read and approved the final manuscript.

## Footnotes

1
http://www.phenoscanner.medschl.cam.ac.uk/phenoscanner

2
www.r-project.org
