# Supplementary material for: Iron status and mental disorders: A Mendelian randomization study
Source: Front Nutr. 2022 Dec 15;9:1084860. doi: 10.3389/fnut.2022.1084860 (PMC9797506; doi:10.3389/fnut.2022.1084860)
Supplement: Supplementary file 1 [file Table_1.DOCX]

**Table S1. Genetic instruments for biomarkers of systemic iron status**

| **SNP** | **Nearby Gene** | **EA** | **EAF** | **Iron (μmol/L)** | | | **Log_10_ Ferritin (μg/L)** | | | **Transferrin saturation (%)** | | | **Transferrin (g/L)** | | |
| --- | --- | --- | --- | --- | --- | --- | --- | --- | --- | --- | --- | --- | --- | --- | --- |
|  |  |  |  | **β** | **SE** | ***P*** | **β** | **SE** | ***P*** | **β** | **SE** | ***P*** | **β** | **SE** | ***P*** |
| rs1800562 | HFE | A | 0.067 | 0.328 | 0.016 | 2.72×10^−97^ | 0.204 | 0.016 | 1.54×10^-38^ | 0.577 | 0.016 | 2.19×10^−270^ | -0.479 | 0.016 | 8.90×10^−196^ |
| rs1799945 | HFE | G | 0.150 | 0.189 | 0.010 | 1.10×10^−81^ | 0.065 | 0.010 | 1.71×10^-10^ | 0.231 | 0.010 | 5.13×10^−109^ | -0.114 | 0.010 | 9.36×10^−30^ |
| rs855791 | TMPRSS6 | G | 0.554 | 0.181 | 0.007 | 1.32×10^−139^ | 0.055 | 0.007 | 1.38×10^-14^ | 0.190 | 0.008 | 6.41×10^−137^ | -0.044 | 0.007 | 1.98×10^−9^ |

EA, nucleotide present at the effect allele; EAF, effect allele frequency; HFE, gene encoding the hemochromatosis protein; SNP, single-nucleotide polymorphism; TMPRSS6, gene encoding transmembrane serine protease 6.
