# Supplementary material for: Iron status and mental disorders: A Mendelian randomization study
Source: Front Nutr. 2022 Dec 15;9:1084860. doi: 10.3389/fnut.2022.1084860 (PMC9797506; doi:10.3389/fnut.2022.1084860)
Supplement: Supplementary file 2 [file Table_2.DOCX]

**Table S2. Study characteristics for included mental disorders.**

| **Disease** | **Phecode** | **ICD-10** | **Cases, No.** | **Controls, No.** | **Population** |
| --- | --- | --- | --- | --- | --- |
| Alzheimer’s disease | 290.11 | G30 | 921 | 410833 | EUR |
| Aphasia | 292.1 | R47.0 | 2325 | 410833 | EUR |
| Mild cognitive impairment | 292.2 | G31.84 | 366 | 410833 | EUR |
| Memory loss | 292.3 | R41.3 | 1036 | 410833 | EUR |
| Hallucinations | 292.6 | R44.3 | 512 | 410833 | EUR |
| Schizophrenia | 295.1 | F20 | 913 | 376046 | AFR,EUR |
| Bipolar disorder | 296.1 | F31 | 1480 | 369930 | EUR |
| Depression | 296.2 | F32 | 596 | 369930 | EUR |
| Suicide or self-inflicted injury | 296.22 | X60-84 | 541 | 369930 | EUR |
| Anxiety disorder | 297.2 | F41 | 2149 | 369930 | EUR |
| Phobia | 300.1 | F40.2 | 11002 | 3842355 | AFR,CSA,EUR |
| Personality disorders | 300.13 | F60 | 1047 | 369930 | EUR |
| Gender identity disorders | 301 | F64 | 518 | 369930 | EUR |
| Psychogenic disorder | 302 | F45.4 | 438 | 369930 | EUR |
| Somatoform disorder | 303.3 | F45 | 474 | 369930 | EUR |
| Adjustment reaction | 303.4 | F43.2 | 630 | 369930 | EUR |
| Eating disorder | 304 | F50 | 405 | 369930 | EUR |
| Tension headache | 305.2 | G44.2 | 131 | 369930 | EUR |
| Learning disorder | 306.9 | F81 | 442 | 369930 | EUR |
| Substance addiction and disorders | 315.1 | F11-16 | 251 | 419655 | EUR |
| Alcohol-related disorders | 316 | F10 | 7751 | 406789 | AFR,CSA,EUR |
| Tobacco use disorder | 317 | F17 | 18000 | 406789 | AFR,CSA,EUR |
| Sleep disorder | 318 | G47 | 17375 | 406789 | AFR,CSA,EAS,EUR,MID |
| Parkinson's disease | 327 | G20 | 7761 | 429105 | AFR,CSA,EUR,MID |

AFR, African ancestry; CSA, Central/South Asian ancestry; EAS, East Asian ancestry; EUR, European ancestry; ICD-10, International Classification of Diseases, Tenth Revision; MID, Middle Eastern ancestry.
